# A Potential Prognostic Marker for Recognizing VEGF-Positive Hepatocellular Carcinoma Based on Magnetic Resonance Radiomics Signature

**DOI:** 10.3389/fonc.2022.857715

**Published:** 2022-04-04

**Authors:** Tingting Fan, Shijie Li, Kai Li, Jingxu Xu, Sheng Zhao, Jinping Li, Xinglu Zhou, Huijie Jiang

**Affiliations:** ^1^ Department of Radiology, The Second Affiliated Hospital of Harbin Medical University, Harbin, China; ^2^ Department of Interventional Radiology, Harbin Medical University Cancer Hospital, Harbin, China; ^3^ Department of Research Collaboration, Research and Development (R&D) Center, Beijing Deepwise & League of Doctor of Philosophy (PHD) Technology Co., Ltd, Beijing, China; ^4^ Department of Positron Emission Tomography/Computed Tomography (PET/CT) Center, Harbin Medical University Cancer Hospital, Harbin, China

**Keywords:** magnetic resonance imaging, hepatocellular carcinoma, radiomics, VEGF, diagnosis

## Abstract

**Objectives:**

The objective of our project is to explore a noninvasive radiomics model based on magnetic resonance imaging (MRI) that could recognize the expression of vascular endothelial growth factor (VEGF) in hepatocellular carcinoma before operation.

**Methods:**

202 patients with proven single HCC were enlisted and stochastically distributed into a training set (n = 142) and a test set (n = 60). Arterial phase, portal venous phase, balanced phase, delayed phase, and hepatobiliary phase images were used to radiomics features extraction. We retrieved 1906 radiomic features from each phase of every participant’s MRI images. The F-test was applied to choose the crucial features. A logistic regression model was adopted to generate a radiomics signature. By combining independent risk indicators from the fusion radiomics signature and clinico-radiological features, we developed a multivariable logistic regression model that could predict the VEGF status preoperatively through calculating the area under the curve (AUC).

**Results:**

The entire group comprised 108 VEGF-positive individuals and 94 VEGF-negative patients. AUCs of 0.892 (95% confidence interval [CI]: 0.839 - 0.945) in the training dataset and 0.800 (95% CI: 0.682 - 0.918) in the test dataset were achieved by utilizing radiomics features from two phase images (8 features from the portal venous phase and 5 features from the hepatobiliary phase). Furthermore, the nomogram relying on a combined model that included the clinical factors α-fetoprotein (AFP), irregular tumor margin, and the fusion radiomics signature performed well in both the training (AUC = 0.936, 95% CI: 0.898-0.974) and test (AUC = 0.836, 95% CI: 0.728-0.944) datasets.

**Conclusions:**

The combined model acquired from two phase (portal venous and hepatobiliary phase) pictures of gadolinium-ethoxybenzyl-diethylenetriamine-pentaacetic acid (Gd-EOB-DTPA)-enhanced MRI could be considered as a credible prognostic marker for the level of VEGF in HCC.

## Introduction

Hepatocellular carcinoma (HCCs) is the most common primary malignant tumor of the liver, with a high mortality rate worldwide (1, 2). Surgical excision is the preferable treatment for HCC, but the relapse and metastasis rates are still high. The Barcelona Clinic Liver Cancer (BCLC) staging system may help with prognosis evaluation and therapy programs (3). However, clinical outcomes can vary greatly among patients within the same BCLC stage because of its molecular heterogeneity. Therefore, the management of HCC is challenging, and understanding the tumor molecular heterogeneity of HCC is important to help develop and optimize therapeutic protocols.

A number of molecular biomarkers have been suggested as potential prognostic factors for poor outcome in HCC, including biomarkers closely associated with cell motility, adhesions, angiogenesis, apoptosis, invasion, etc. (4–7). Among the angiogenesis-related factors, the primary angiogenic factor is vascular endothelial growth factor (VEGF) (8). VEGF, also known as vascular permeability factor, was discovered in 1983 as a protein released by tumor cells. VEGF can promote the formation of fibrin scaffolds and induce new blood vessel sprouting around growing tumors. By interacting with multiple of its receptors (VEGFR 1, VEGFR 2, VEGFR 3), VEGF induces angiogenesis (9). The VEGF family comprises six members (VEGFA-E, and placental growth factor). VEGF-A (often referred to as VEGF) is the most prototypical and the best-studied VEGF family member. The overexpression of VEGF can occur in various human tumors, including HCC (10, 11). Overexpression of VEGF has been associated with the development and progression of HCC in previous studies (12). Immunosuppression caused by VEGF inside the malignancy and its surroundings can be reduced by anti-VEGF therapies (13). A successful phase III trial (IMbrave150) that assessed the combination of atezolizumab and bevacizumab versus sorafenib demonstrated a marked improvement in clinical outcomes for advanced hepatocellular carcinoma (14). Anti-VEGF therapy plays an important role in the therapeutic drugs for HCC. The difference in VEGF expression level will affect the treatment efficacy. Previous studies revealed that higher VEGF levels were associated with more invasive disease, shorter survival times, and worse results following surgery and local treatment (15). So it is necessary to investigate the VEGF expression. From this perspective, it appears that the analysis of VEGF expression may enhance the clinical management of HCC. Hence, early diagnosis of VEGF-positive HCC is the key to choosing the best optimal treatment strategy.

Radiomics is a new domain in medicine that utilizes high-throughput quantitative image features based on images from imaging examinations that cannot be resolved by the naked eye for diagnosis and prognosis (16). These features potentially capture the intratumoral heterogeneity, which can offer information about the tumor microenvironment and the phenotype of cancer (17). The strengths of multi-parametric magnetic resonance imaging (MRI) are high temporal resolution, excellent soft tissue definition, and lack of ionizing radiation in tumor imaging (18). Some studies reported that the radiomics signature derives from contrast-enhanced MRI could be used as the imaging biomarker in the microvascular invasion and Cytokeratin19 status of HCC (19, 20). However, as far as we know, few research concentrates on the prediction of VEGF-positive HCC based on MRI radiomics signature. Thus, this project was targeted to analyze the feasibility of multi-parametric MRI radiomics models for predicting VEGF-positive HCC.

## Materials and Methods

### Patients and MRI Protocol

**Figure 1:**
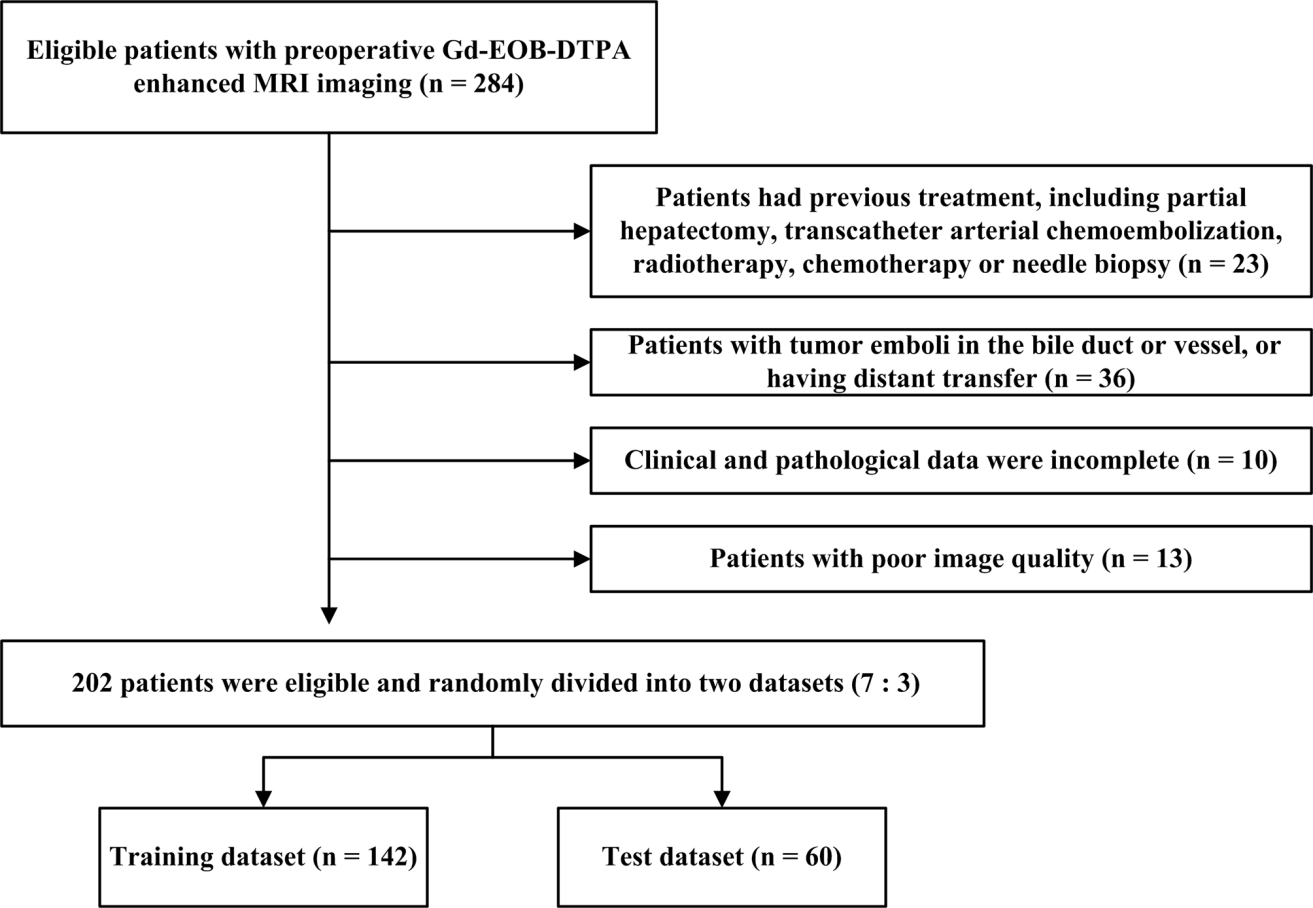
Patient selection flow chart.

The present research met with the approval of our Hospital Review Board (approval number KY2019-217) and written informed assent was exempted for all participators. The participants with HCC were enrolled from imaging database management system between March 2017 and March 2021. Gadolinium-ethoxybenzyl-diethylenetriamine-pentaacetic acid (Gd-EOB-DTPA, Primovist, Germany)-enhanced MRI imaging was carried out for a total of 284 patients who were suspected of HCC and experienced subsequent hepatic excision. The subject entry criteria were as follows: 1) Patients had a definite diagnosis of single primary HCC by pathology; 2) spanning less than 14 days between the first MRI examination and surgery. The exclusion criteria for patients included: 1) Patients had previous treatment, including partial hepatectomy, transcatheter arterial chemoembolization (TACE), radiotherapy, chemotherapy or needle biopsy (n = 23); 2) patients with tumor emboli in the bile duct or vessel (n = 24), or having distant transfer (n = 12); 3) Clinical and pathological data were incomplete (n = 10); 4) with poor image quality (n = 13). Finally, 202 patients (169 men and 33 women) were eligible and randomly divided into two datasets at a proportion of 7:3. Details of baseline, involving age, gender, tumor diameter, etiology of liver disease, cirrhosis, ascites, aspartate aminotransferase (AST), alanine aminotransferase (ALT), total bilirubin (TBIL), neutrophil count, lymphocyte count, neutrophil-to-lymphocyte ratio (NLR), albumin, serum α-fetoprotein (AFP), differentiation degree, were derived from medical records. **Supplementary Method 1** presents the exhaustive MRI procedure. The flowchart for patient selection is demonstrated in **Figure 1**.

### Immunohistochemical Analysis of VEGF

All patients underwent subsequent radical resection of HCC. The detailed immunohistochemical protocol is explained in **Supplementary Method 2**. Two observers without knowledge of the clinicopathologic parameters scored the slides independently using a previously validated scoring system. Any disagreements were arbitrated by a third rater. VEGF scoring was calculated using the immune reactive score (IRS) as depicted before (21–23): IRS = SI (staining intensity) × PP (percentage of positive cells). SI was graded as 0(negative), 1 (weak), 2 (moderate) and 3 (strong). PP was categorized as 0 (negative), 1 (less than 5% positive cells), 2 (6-25% positive cells), 3 (26-50% positive cells), 4 (51-75% positive cells), and 5 (more than 75% positive cells). The IRS was evaluated using ten visual fields from different regions of each tumor, and the average IRS was determined. The IRS classified VEGF status into four categories: grade 0 (IRS 0), grade 1 (IRS 1-4), grade 2 (IRS 5-9), and grade 3 (IRS 10-15). VEGF (grade 0 and grade 1) was defined as VEGF-negative expression, while VEGF (grade 2 and grade 3) was marked as VEGF-positive expression (24).

### Morphologic Features

Two radiologists who were both lack of the histopathologic information analyzed the MR images on a Picture Archiving and Communication System (PACS) work-station. They negotiated every divergence to make a consensus. **Supplemental Table 1** demonstrated the agreements of inter-observer about morphologic MRI features. The following qualitative morphologic variables were evaluated (20): (a) The tumor margin was classified as either smooth or irregular. It was defined irregular tumor margin if the ill-defined interface with normal tissue on HBP images was present; (b) arterial rim enhancement, described as a ring-enhanced with central low signal areas on arterial phase; (c) radiological capsule presence or absence.

### HCC Segmentation

All images of the HCC tumors were uploaded on the online Deepwise Research Platform (https://research.deepwise.com). Radiologist A, who was ignorant of the pathological conditions and had five years of expertise interpreting HCC imaging, delineated the HCC lesions. Radiologist B validated the region of interests (ROIs). ROIs were individually delineated on pictures from the arterial phase (AP), portal venous phase (PVP), balanced phase (BP), delayed phase (DP), and hepatobiliary phase (HBP). Twenty-two HCCs were arbitrarily chosen and re-segmented one week later by the two radiologists, in order to calculate the intra-class and inter-class correlation coefficient (ICCs).

### Clinical and Morphologic Risk Features

We used logistic regression (univariate and multivariate) to evaluate the connection between basic information, morphologic features and VEGF expression in HCC of the training dataset. The stopping rule was a backward stepwise selection based on Akaike’s information criterion and the likelihood ratio test. In the multivariate analysis, the potential risk factors were selected as those with a p value < 0.05, and the clinical model derived from these variables was established for the training dataset.

### Radiomic Feature Analysis

The radiomics analysis technique involved ROI (HCCs) segmentation, feature extraction, feature selection, model building, and testing. The Python package Pyradiomics (Version 3.0.1, https://github.com/Radiomics/pyradiomics) was used for feature extraction (25). The following four steps presented the technique of feature extraction and selection in detail.

1. Standardization of spacing: All MRI pictures were resampled to pixel spacing of 2.0 × 2.0 × 2.0 mm with the aid of interpolator of sitkBSpline in the Python package SimpleITK. This step can eliminate interference from various scales (26).
2. Image filtering and feature calculation: The raw MRI images were processed by a wavelet filter and a LaPlacian-Gaussian filter to strengthen the differentiation of features. The shape-based characteristics, first-order and texture features were calculated and studied in present study. Eventually, 1906 radiomics features were obtained from every MRI phase for each lesion. The Z-score was standardized for the extracted features.
3. ICC test: The reproducible features were set as the features with an intra- and inter-observer ICC greater than 0.8 (27). These robust features were applied in the following feature selection.
4. Feature selection: The linear correlation coefficient between characteristics was initially estimated, and one of them was deleted when it was ≥ 0.65. Furthermore, we applied the analysis of variance (ANOVA) F-test to each feature and label pair to conduct feature selection. Based on the F-value ranking, the top 14 features were adopted as meaningful predictors in the subsequent logistic regression.

### Model Building

For additional radiomics signature development, logistic regression was used for radiomics characteristics determined by the F-test. For the optimal parameter selection in the training dataset, we utilized ten-fold cross-validation. The radiomics signature was created *via* the parameters that produced the best average area under the curve (AUC). The phases with AUCs larger than 0.700 were judged noteworthy and hence were chosen for the development of fusion radiomics signatures.

A likelihood ratio backward stepwise multivariable logistic regression model that combined clinical factors with radiomics signature was constructed to investigate whether these two variables enhanced prediction of VEGF status. Clinical variables with a significant correlation with VEGF, as well as radiomics signatures, were added into the multivariable combined model. The discriminatory ability of classifier model was analyzed by the AUCs, and the Delong test was used to compare AUCs. The Hosmer-Lemeshow test was used to determine the calibration performance between the combined model’s projected probability and the actual VEGF status.

**Figure 2:**
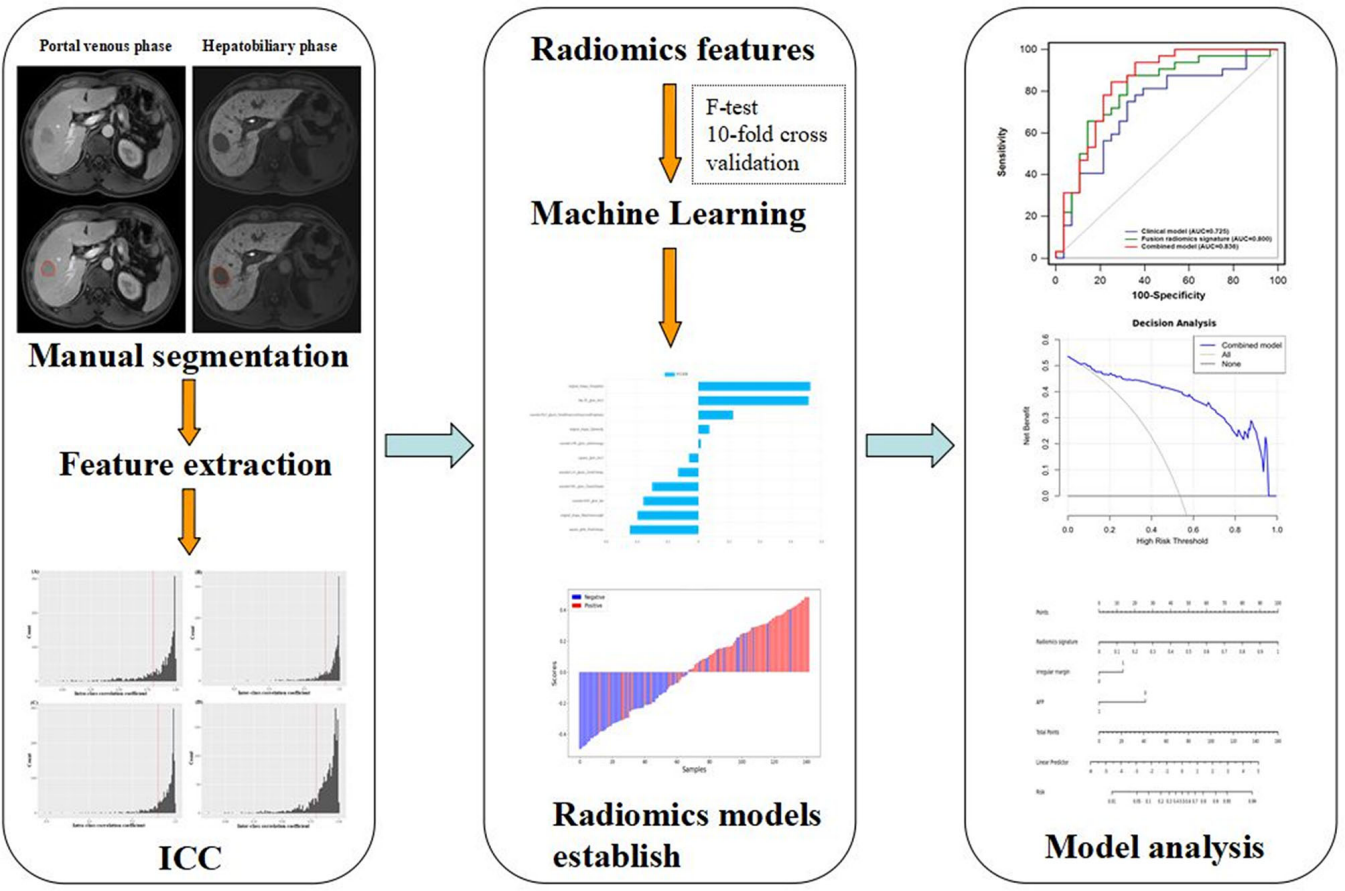
Diagram of HCC lesions segmentation, feature extraction, ICC test, feature selection, and model establish and analysis.

Furthermore, we established a nomogram relying on a combined model that can simply calculate the likelihood of VEGF status. The clinical utility of the nomogram was appraised by means of decision curve analysis (28). By measuring the disparity between the true- and false-positive rate at different thresholds, net benefits were calculated (29). **Figure 2** describes a diagram of a radiomics investigation.

### Statistical Analysis

We used Student’s t-test or Mann-Whitney U test for continuous variables and Chi-Square test or Fisher’s exact test for qualitative variables in training and test datasets. The statistical analyses were carried out using IBM SPSS Statistics 22.0 and MedCalc 20 software (MedCalc Software, Belgium). Statistical significance was defined as a two-tailed P-value < 0.05.

## Results

### Clinicopathologic Characteristics and Clinical Modeling

In **Table 1**, demographics, laboratory results, and morphological characteristics of both the training and tested groups are summarized and compared. 202 individuals (169 male and 33 female) with solitary HCC were split into a training group (n = 142, 76 for VEGF positive, 66 for VEGF negative; 115 male and 27 female) and a testing group (n = 60, 32 for VEGF positive, 28 for VEGF negative; 54 male and 6 female).

**Table 1:** Patient characteristics in the training and test cohorts.

Characteristics	Training dataset (n = 142)		P value	Test dataset (n = 60)		P value	P value*
	VEGF(+) (n = 76)	VEGF(-) (n = 66)		VEGF(+) (n = 32)	VEGF(-) (n = 28)		0.980
Age, mean ± SD, years	55.72 ± 8.12	53.97 ± 9.73	0.244	56.34 ± 7.12	53.32 ± 9.60	0.168	0.809
Gender, n (%)			0.847			0.796	0.113
Male Female	62 (81.6)	53 (80.3)		28 (87.5)	26 (92.9)		
	14 (18.4)	13 (19.7)		4 (12.5)	2 (7.1)		
Diameter, median (IQR), mm	35.50 (27.50,49.75)	46.00 (27.00,61.00)	0.067	44.00 (30.25,55.75)	46.00 (34.75,49.75)	0.876	0.401
Etiology of liver disease			0.436			0.377	0.400
HBV positive ^a^	67 (88.2)	61 (92.4)		28 (87.5)	22 (78.6)		
HCV positive ^b^	1 (1.3)	0 (0)		0 (0)	1 (3.6)		
None or other	8 (10.5)	5 (7.6)		4 (12.5)	5 (17.9)		
Cirrhosis, n (%)			0.184			0.796	0.613
Present Absent	55 (72.4)	54 (81.8)		26 (81.3)	22 (78.6)		
	21 (27.6)	12 (18.2)		6 (18.8)	6 (21.4)		
Ascites, n (%)			0.995			0.830	0.167
Present	15 (19.7)	13 (19.7)		4 (12.5)	3 (10.7)		
Absent	61 (80.3)	53 (80.3)		28 (87.5)	25 (89.3)		
ALT (U/L)	28.95 (16.25,45.68)	33.00 (21.25,45.63)	0.305	29.00 (18.25,52.50)	28.50 (17.25,46.50)	0.583	0.793
AST (U/L)	30.00 (21.25,40.00)	30.00 (24.00,56.25)	0.112	37.00 (21.50,54.50)	29.00 (20.25,51.25)	0.320	0.901
TBIL (μmol/L)	15.15 (11.25,20.57)	14.25 (11.40,18.45)	0.756	14.30 (11.90,18.40)	16.25 (10.48,21.85)	0.543	0.817
Neutrophil count (×10^9^/L)	2.75 (1.95,3.48)	3.07 (2.20,3.88)	0.081	2.41 (1.88,3.39)	3.00 (2.03,4.01)	0.251	0.737
Lymphocyte count (×10^9^/L)	1.79 ± 0.63	1.68 ± 0.67	0.315	1.97 ± 0.74	1.86 ± 0.70	0.543	0.277
NLR	1.53 (1.10, 2.01)	1.75 (1.28, 2.61)	0.029	1.30 (0.88, 1.84)	1.73 (1.45, 2.12)	0.105	0.224
Albumin (g/L)	37.86 ± 4.17	37.62 ± 3.74	0.720	38.18 ± 3.17	37.58 ± 2.90	0.450	0.769
Serum AFP, n (%)			0.001			0.352	0.065
≤400 ng/mL	66 (86.8)	42 (63.6)		22 (68.8)	16 (57.1)		
>400 ng/mL	10 (13.2)	24 (36.4)		10 (31.3)	12 (42.9)		
Differentiation degree, n (%)			0.166			0.722	0.208
Well	12 (15.8)	15 (22.7)		5 (15.6)	5 (17.9)		
Moderate	51 (67.1)	44 (66.7)		19 (59.4)	17 (60.7)		
Poor	13 (17.1)	7 (10.6)		8 (25.0)	6 (21.4)		
**Morphologic MR features**							
Irregular margin on HBP, n (%)			0.015			0.001	0.336
Absence Presence	25 (32.9)	35 (53.0)		5 (15.6)	16 (57.1)		
	51 (67.1)	31 (47.0)		27 (83.4)	12 (42.9)		
Arterial rim enhancement, n (%)			0.309			0.726	0.245
Presence Absence	48 (63.2)	47 (71.2)		18 (56.3)	17 (60.7)		
	28 (36.8)	19 (28.8)		14 (43.8)	11 (39.3)		
Tumor capsule, n (%)			0.879			0.821	0.793
Complete Incomplete or absent	55 (72.4)	47 (71.2)		22 (68.8)	20 (71.4)		
	21 (27.6)	19 (28.8)		10 (31.3)	8 (28.6)		
Enhancement pattern, n (%)			0.678			0.768	0.713
Arterial enhancement with washout	62 (81.6)	52 (78.8)		26 (81.3)	25 (89.3)		
No or minimal enhancement	6 (7.9)	9 (13.6)		3 (9.4)	1 (3.6)		
Persistent enhancement	6 (7.9)	4 (6.1)		2 (6.3)	1 (3.6)		
Progressive enhancement	2 (2.6)	1 (1.5)		1 (3.1)	1 (3.6)		

VEGF (+), VEGF-positive; VEGF (−), VEGF-negative; ALT, alanine aminotransferase; AST, aspartate aminotransferase; TBIL, total bilirubin; NLR, neutrophil-to-lymphocyte ratio; AFP, α-fetoprotein; HBP, hepatobiliary phase; SD, standard deviation; IQR, interquartile range. *Represents the comparisons of characteristics between training and test dataset. Data are mean ± SD, median (IQR) or n (%), where n is the number of participants for whom data is available. ^a^Represents positivity for hepatitis B serum antigen. ^b^Represents positivity for serum HCV antibody.

Across the full cohort, there were 108 (53.5%) patients who were VEGF-positive and 94 (46.5%) patients who were VEGF-negative. VEGF status was not significantly different between the training and test datasets (p = 0.980). In the training cohort, univariate analysis revealed that serum ɑ-fetoprotein (AFP), irregular margin, and neutrophil-to-lymphocyte ratio (NLR) were significantly associated with VEGF (P < 0.05) (**Table 1**). However, age, gender, tumor diameter, etiology of liver disease, cirrhosis, ascites, ALT, AST, TBIL, neutrophil count, lymphocyte count, albumin, differentiation degree, arterial rim enhancement, tumor capsule, and enhancement pattern were not statistically significant (P > 0.05). A backward stepwise multivariable logistic regression was used to examine every notable variable. Eventually, the meaningful variables for clinical model establishing were serum AFP level (odds ratio [OR] = 0.260; 95% confidence interval [CI]:0.107-0.633, P = 0.003), irregular tumor margin (p = 0.004, OR = 3.004, 95% CI: 1.434 - 6.295), and NLR (p = 0.027, OR = 0.622, 95% CI: 0.409 - 0.948) (**Table 2**). In the training cohort, the clinical model’s AUC was 0.709 (95% CI: 0.624 - 0.794) while it was 0.725 (95% CI: 0.593 - 0.858) in the test cohort.

**Table 2:** Univariate and multivariate assessments of variables associated with VEGF levels in clinical model.

	Univariate		Multivariate	
	OR (95%CI)	P value	OR (95%CI)	P value
Age	1.023 (0.985 - 1.062)	0.243		
Gender	0.921 (0.398 - 2.131)	0.847		
NLR	0.625 (0.423 - 0.924)	0.019	0.622 (0.409 - 0.948)	0.027
Irregular margin on HBP	2.303 (1.167- 4.547)	0.016	3.004 (1.434 - 6.295)	0.004
Serum AFP level	0.265 (0.115 - 0.610)	0.002	0.260 (0.107 - 0.633)	0.003

AFP, α-fetoprotein; NLR, neutrophil-to-lymphocyte ratio; CI, confidence interval; HBP, hepatobiliary phase; OR, odds ratio.

### Feature Selection and Radiomics Model Development

The features generated from arterial phase, PVP, balanced phase, delayed phase, and HBP were decreased to 1349, 1252, 1850, 1816, and 1202, respectively, after feature selection through the ICC test. Those robust features were applied for further analysis. **Table 3** displays the forecast results for each of the five phases. Finally, the test set’s median 10-fold cross-validation AUC for PVP, HBP, AP, BP, and DP was 0.759, 0.731, 0.640, 0.592, and 0.692, respectively. The radiomics signatures of PVP and HBP (> 0.700) were captured to investigate further. **Figure S1** presents the stability of radiomics characteristics in two phases (PVP and HBP) images. After F-test modeling for the prediction of VEGF-positive, 8 robust features in the PVP and 5 robust features in the HBP were regarded as the optimal feature set. The selected features were then used to create a logistic regression classifier model. **Tables S2**, **S3** provide more detailed information on the features. In the training cohort, the radiomics signature in the PVP obtained an AUC of 0.809 (95% CI: 0.740 - 0.878) while in the test dataset, it had an AUC of 0.759 (95% CI: 0.630 - 0.888). Likewise, in the training and test groups, the radiomics signature of HBP obtained an AUC of 0.792 (95% CI: 0.716 - 0.868) and 0.731 (95% CI: 0.597 - 0.865), respectively.

**Table 3:** Forecasting results of the clinical model, radiomics model and the combined model.

Model	Training dataset (n = 142)							Test dataset (n = 60)						
	AUC (95% CI)	PNP	SENS	SPEC	ACC	PPV	NPV	AUC (95% CI)	PNP	SENS	SPEC	ACC	PPV	NPV
Clinical model	0.709 (0.624-0.794)	92	0.684	0.606	0.648	0.667	0.625	0.725 (0.593-0.858)	41	0.656	0.714	0.683	0.724	0.645
Radiomics model of different MRI phase														
AP	0.709 (0.622-0.797)	98	0.711	0.667	0.690	0.711	0.667	0.640 (0.497-0.782)	37	0.531	0.714	0.617	0.680	0.571
PVP	0.809 (0.740-0.878)	99	0.724	0.667	0.697	0.714	0.677	0.759 (0.630-0.888)	38	0.625	0.643	0.633	0.667	0.600
BP	0.748 (0.666-0.830)	96	0.658	0.697	0.676	0.714	0.639	0.592 (0.437-0.746)	32	0.438	0.643	0.533	0.583	0.500
DP	0.668 (0.578-0.757)	87	0.618	0.606	0.613	0.644	0.580	0.692 (0.554-0.830)	35	0.406	0.786	0.583	0.684	0.537
HBP	0.792 (0.716-0.868)	109	0.763	0.773	0.768	0.795	0.739	0.731 (0.597-0.865)	39	0.500	0.821	0.650	0.762	0.590
PVP + HBP	0.892 (0.839-0.945)	120	0.803	0.894	0.845	0.897	0.797	0.800 (0.682-0.918)	43	0.594	0.857	0.717	0.826	0.649
Combined model	0.936 (0.898-0.974)	123	0.855	0.879	0.866	0.890	0.841	0.836 (0.728-0.944)	43	0.625	0.821	0.717	0.800	0.657

ACC, accuracy; AP, arterial phase; AUC, area under curve; BP, balanced phase; HBP, hepatobiliary phase; DP, delayed phase; SPEC, specificity; SENS, sensitivity; NPV, negative predictive value; PNP, predicted number of patients correctly classified; PPV, positive predictive value; PVP, portal venous phase; PVP + HBP, a fusion radiomics model integrating the hepatobiliary and portal venous phases.

Furthermore, we propose a multiphase fusion model that combines the radiomics signatures (PVP and HBP) with a multivariable logistic regression model to study the maximum potential utilization of both radiomics signatures in diverse MRI phases. The fusion radiomics signature performed well in training and test groups, with AUCs of 0.892 (95% CI: 0.839 - 0.945) and 0.800 (95% CI: 0.682 - 0.918), respectively. When compared to the clinical model, the difference was significant in the training (p = 0.0003) cohort; however, the p value in the test dataset was 0.3759. We also noticed substantial variations in fusion signatures between VEGF (+) and VEGF (-) patients, and the p values of both (training and test) groups were less than 0.001.

### Combined Model Building

The combined model performed best for VEGF prediction, combining all potential parameters including the fusion radiomics signature and major clinical characteristics (irregular tumor margin and serum AFP levels). Compared with the clinical model, the combined model represents superior performance in training [0.936(0.898 - 0.974) vs. 0.709(0.624-0.794); p < 0.0001] and test [0.836(0.728-0.944) vs. 0.725(0.593-0.858); p = 0.093] cohorts, albeit the difference was not statistically meaningful (p = 0.093) in test datasets. Furthermore, the accuracies of the combined model for forecasting the VEGF were 86.6% (training group) and 71.7% (test group) (**Table 3**).

**Figure 3:**
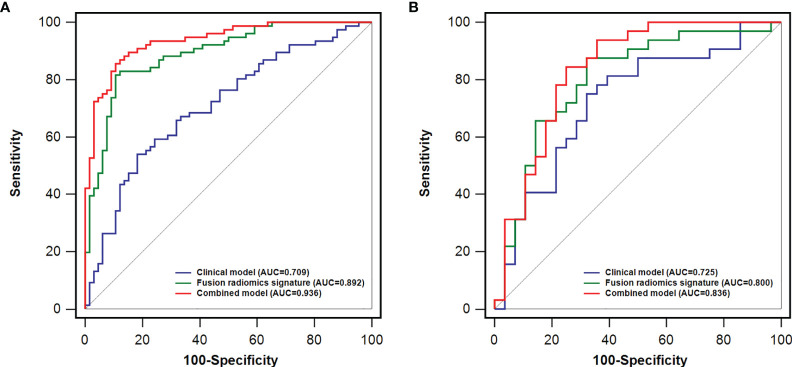
A comparison of receiver operating characteristics (ROC) curves for predicting HCC VEGF status. ROC curves of clinical factors, the fusion radiomics signature, and the combined model in the training **(A)** and test **(B)** dataset.

**Figure 4:**
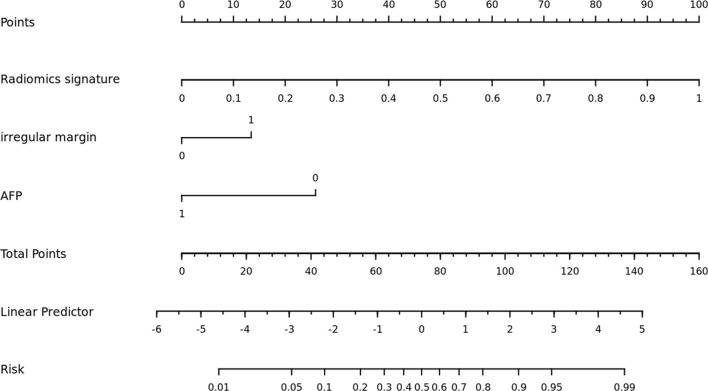
The nomogram was built using a combination model that included radiomics signature, irregular margin, and serum AFP level.

**Figure 5:**
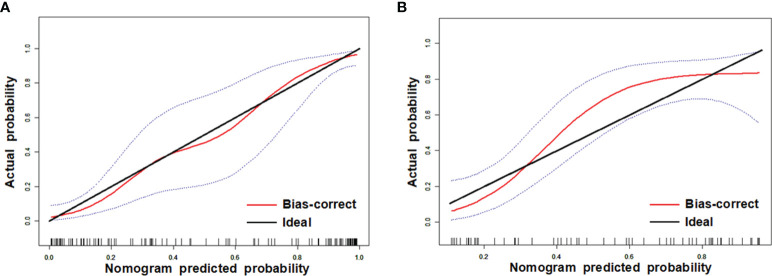
Calibration curves of training **(A)** and test **(B)** datasets. The y axis shows the patients’ real VEGF positivity rate, while the x axis shows the nomogram-forecasted likelihood of VEGF positivity. The black slant solid line denotes a faultless agreement as determined by an ideal model. The blue dashed lines represent 95% confidence interval [CI].

**Figure 6:**
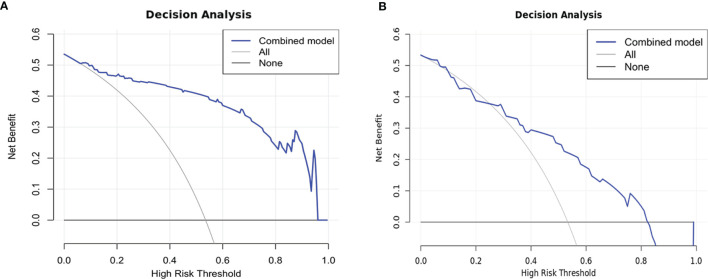
Decision curves of training **(A)** and test **(B)** datasets. The net benefit is shown on the y-axis, while the threshold probability is represented on the x-axis. The blue line represents the combined model’s benefit. The grey and black lines depict the tactics of “treating everyone” and “treating none”, respectively.

Comparisons among AUCs between every two model were showed in **Table S4**. The stratified analysis also revealed strong results in both age and gender categories (**Table S5**). **Figure 3** displays the three ROC curves for different models. **Figure 4** depicts the created nomogram. The calibration curves are displayed in **Figure 5**, and there is high conformity in the two groups (training: P = 0.820; test: P = 0.508). **Figure 6** depicts the findings of the decision curve analysis (DCA). The DCA for combined model indicated that when the probability of VEGF-positive was between 0.060-0.976 and 0.250-0.823 in the training and test datasets, respectively, using the nomogram to evaluate pathological VEGF-positive had added benefit compared with treating all patients as positive or negative. The coefficients and relative weights of 13 selected radiomics features of logistic regression in combined model are displayed on **Table S6**. A correlation heat map of the selected 13 radiomics features in the combined model is depicted on **Figure S2**.

## Discussion

To detect VEGF-positive HCC preoperatively, we created a predictive nomogram using MRI (Gd-EOB-DTPA-enhanced) radiomics signatures, serum AFP levels, and irregular tumor margins. The combined model revealed adequate diagnostic capacity in stratifying HCCs based on VEGF status. The fusion radiomics signature outperformed radiomics signatures derived independently in PVP or HBP, with AUCs of 0.892 in training and 0.800 in test datasets for detecting VEGF-positive HCCs. The combined model attained the greatest AUC of 0.936 in the training cohort and an AUC of 0.836 in the test cohort after adding clinical risk factors and fusion imaging biomarkers. Previous research discovered that high levels of VEGF in HCC lesions were substantially related to metastatic recurrence (30). So far as we know, though, no studies have been published that have utilized a nomogram to forecast the likelihood of VEGF positivity in HCC.

In this study, radiomic features of the three-dimensional (3D) primary tumor were utilized to predict VEGF-positive HCC. The 3D tumor volume may give more precise tumor heterogeneity information as well as full size and shape information (31, 32). Qualitative and quantitative analysis using Gd-EOB-DTPA-enhanced MRI imaging can help characterize VEGF-positive HCCs. Despite the availability of different MRI sequences, only 8 and 5 radiomics characteristics in PVP and HBP images were retrieved for the greatest prediction performance. In our single-phase study, the predictive performance of the model for the PVP or HBP was better than that for the AP. Of interest, it has been previously reported that the same two phases (PVP and HBP) pictures of Gd-EOB-DTPA-enhanced MRI may be for the diagnosis of early HCC when arterial enhancement lack (33). Their results suggest that changes in hepatocyte function may precede significant vascular changes in early hepatocellular carcinoma. It could be speculated that such a pattern may be related to the development of subsequent arterial hyperenhancement. To our knowledge, VEGF expression in hepatocyte precedes the changes in angiogenesis in the tumor microenvironment. The low diagnostic performance of the radiomics model obtained in the AP may be due to the immaturity of tumor vasculature. PVP and HBP may reflect the functional changes of hepatocytes earlier than AP, so that we can find more information related to hepatocyte function from the characteristics and then better predict VEGF expression than in AP. Furthermore, we discovered two features, “wavelet-LLH_firstorder_Range” and “square_glrlm_RunEntropy” that corresponded to the biological characteristics. “Wavelet-LLH_ firstorder_Range represents the range of gray values in the ROI which is derived from imaging after wavelet filtering. “Square_glrlm_RunEntropy” quantifies the degree of uncertainty or randomness in the distribution of run lengths and gray levels produced from imaging after square filtering. A greater value suggests that the texture patterns are more heterogeneous. Intratumoral heterogeneity may be related to the difference between VEGF positive and VEGF negative HCC (e.g., angiogenesis, invasion, and apoptosis) (34–36).

There was no considerable difference in the clinicopathologic characteristics of VEGF-positive HCCs between the training and test populations. In existing researches (30, 37), the value of VEGF expression correlated positively with tumor size and AFP level in tumor tissue. However, in our study, there was no effect of tumor size on VEGF expression in HCCs. This may relate to the sample size and sampling strategy. Although there were no significant statistical differences in our study, VEGF-positive HCCs were more prone to having an invasive character, defined as low-differentiated and bigger tumor size (30, 38). Preoperative serum AFP level may be used as a predictive marker in the present study. In clinical practice, preoperative serum AFP level is readily available.

Our findings indicated that an irregular margin of tumor was closely tied with VEGF expression. The irregular tumor margin of preoperative MRI imaging features could identify high-risk early recurrence patients with early-stage hepatocellular carcinoma (39). The more irregular the margin, the greater the surface region; therefore, it may demonstrate the aggressiveness of the tumor (40). The invasive edge is a notable location for tumor angiogenesis activation, which leads to tumor invasion and metastasis (41).

The combined model, which included a radiomics signature and clinical risk indicators, significantly increased performance. This showed that radiomics signatures and clinical risk factors may be used in tandem. Finally, for clinical application, we created a nomogram, which was a simple tool for calculating the likelihood of VEGF positivity. Patients may be classified as low-risk or high-risk based on the nomogram’s predicted likelihood. A previous study revealed that bevacizumab can target VEGF isoforms and inhibit their interaction with the VEGF receptor (15). In particular, it has been recently reported that among the tyrosine kinase inhibitors (TKI), regorafenib is a drug targeting all of the VEGF receptors (VEGFR1-3) (42). The application of these targeted anti-angiogenesis drugs to patients with a high expression level of VEGF can effectively inhibit angiogenesis in patients with HCC. And therefore this proposed nomogram could help clinicians choose effective systemic treatment options for cancer management. It can not only eliminate unnecessary medical care and expenditures for low-risk individuals, but also significantly minimize patient side effects.

Certainly, some limitations of this study deserve to be mentioned. Firstly, because this was a retrospective investigation, several confounding variables may have existed. Secondly, as this was a single-centre study. Therefore, our results need to be tested by multicenter studies. Thirdly, the genetic factors associated with VEGF were not included, which might give valuable new information for VEGF prediction. Finally, it is worth noting that our study did not include multimodal radiological data. In the future, MRI, computed tomography (CT), ultrasonography (US), and positron emission tomography (PET) data may be used to investigate the status of VEGF.

## Conclusion

HBP and PVP radiomics signatures obtained from Gd-EOB-DTPA-enhanced MRI imaging could assist in predicting the VEGF levels of HCC. In the VEGF stratification of HCC, a forecasting nomogram integrating MRI radiomics characteristics, irregular tumor margins, and serum AFP levels revealed considerably enhanced diagnostic performance.

## Data Availability Statement

The raw data supporting the conclusions of this article will be made available by the authors, without undue reservation.

## Ethics Statement

The studies involving human participants were reviewed and approved by The Second Affiliated Hospital of Harbin Medical University. Written informed consent for participation was not required for this study in accordance with the national legislation and the institutional requirements. 

## Author Contributions

TF and HJ came up with the idea, carried out the experiments, gathered and evaluated the data, conducted the analysis, and drafted the paper. TF, SL, KL, HJ, and JX conducted experiments and/or gathered information. The paper was modified by SZ, HJ, JL, and XZ. The essay was written by all of the writers, and the final version was approved by all of them.

## Funding

Our study has received funding by National Key Research and Development Program of China (2019YFC0118100), National Natural Science Foundation of China (No. 81671760, 81873910, 62171167).

## Conflict of Interest

Author JX is employed by Beijing Deepwise & League of PHD Technology Co., Ltd.

The remaining authors declare that the research was conducted in the absence of any commercial or financial relationships that could be construed as a potential conflict of interest.

## Publisher’s Note

All claims expressed in this article are solely those of the authors and do not necessarily represent those of their affiliated organizations, or those of the publisher, the editors and the reviewers. Any product that may be evaluated in this article, or claim that may be made by its manufacturer, is not guaranteed or endorsed by the publisher.

## Abbreviations

AFP, α-fetoprotein; AP, arterial phase; AUC, Area under curve; BCLC, Barcelona Clinic Liver Cancer; BP, balanced phase; CI, confidence interval; CT, computed tomography; DCA, decision curve analysis; DP, delayed phase; Gd-EOB-DTPA, Gadolinium-ethoxybenzyl-diethylenetriamine-pentaacetic; HBP, Hepatobiliary phase; HCC, Hepatocellular carcinoma; ICC, Inter-correlation coefficient; IRS, Immune reactive score; MRI, Magnetic resonance imaging; NLR, Neutrophil-to-lymphocyte ratio; OR, Odds ratio; PACS, Picture Archiving and Communication System; PET, positron emission tomography; PP, Percentage of positive cells; PVP, Portal venous phase; SI, Staining intensity; TACE, Transcatheter arterial chemoembolization; TKI, tyrosine kinase inhibitors; VEGF, Vascular endothelial growth factor.

## References

[1] Wang H Li W. Recent Update on Comprehensive Therapy for Advanced Hepatocellular Carcinoma. World J Gastrointest Oncol (2021) 13:845–55. doi: 10.4251/wjgo.v13.i8.845 PMC8371518 34457190

[2] Ye S Ni Y. lncRNA SNHG9 Promotes Cell Proliferation, Migration, and Invasion in Human Hepatocellular Carcinoma Cells by Increasing GSTP1 Methylation, as Revealed by CRISPR-Dcas9. Front Mol Biosci (2021) 8:649976. doi: 10.3389/fmolb.2021.649976 33898523 PMC8062810

[3] Forner A Reig M Bruix J. Hepatocellular Carcinoma. Lancet (2018) 391:1301–14. doi: 10.1016/S0140-6736(18)30010-2 29307467

[4] Ju G Zhou T Zhang R Pan X Xue B Miao S. DUSP12 Regulates the Tumorigenesis and Prognosis of Hepatocellular Carcinoma. PeerJ (2021) 9:e11929. doi: 10.7717/peerj.11929 34414037 PMC8344690

[5] Qin L Huang D Huang J Huang H. New Biomarkers and Therapeutic Targets of Human Liver Cancer: Transcriptomic Findings. Biofactors (2021) 47:1016–31. doi: 10.1002/biof.1775 34379335

[6] Thomann S Weiler SME Wei T Sticht C de la Torre C Toth M. YAP-Induced Ccl2 Expression Is Associated With a Switch in Hepatic Macrophage Identity and Vascular Remodelling in Liver Cancer. Liver Int (2021) 41:3011–23. doi: 10.1111/liv.15048 34459091

[7] Zhang K Liu H Yu M Zhao H Yang N Bi X. Upregulated LINC01667 Expression Is Correlated With Poor Prognosis in Hepatocellular Carcinoma. Front Oncol (2021) 11:650173. doi: 10.3389/fonc.2021.650173 34458133 PMC8397520

[8] Sun DS Guan CH Wang WN Hu ZT Zhao YQ Jiang XM. LncRNA NORAD Promotes Proliferation, Migration and Angiogenesis of Hepatocellular Carcinoma Cells Through Targeting miR-211-5p/FOXD1/VEGF-A Axis. Microvasc Res (2021) 134:104120. doi: 10.1016/j.mvr.2020.104120 33309645

[9] Kaseb AO Hanbali A Cotant M Hassan MM Wollner I Philip PA. Vascular Endothelial Growth Factor in the Management of Hepatocellular Carcinoma: A Review of Literature. Cancer (2009) 115:4895–906. doi: 10.1002/cncr.24537 19637355

[10] Apte RS Chen DS Ferrara N. VEGF in Signaling and Disease: Beyond Discovery and Development. Cell (2019) 176:1248–64. doi: 10.1016/j.cell.2019.01.021 PMC6410740 30849371

[11] Butt SS Khan K Badshah Y Rafiq M Shabbir M. Evaluation of Pro-Apoptotic Potential of Taxifolin Against Liver Cancer. PeerJ (2021) 9:e11276. doi: 10.7717/peerj.11276 34113483 PMC8162243

[12] Morse MA Sun W Kim R He AR Abada PB Mynderse M. The Role of Angiogenesis in Hepatocellular Carcinoma. Clin Cancer Res (2019) 25:912–20. doi: 10.1158/1078-0432.CCR-18-1254 30274981

[13] Voron T Colussi O Marcheteau E Pernot S Nizard M Pointet AL. VEGF-A Modulates Expression of Inhibitory Checkpoints on CD8+ T Cells in Tumors. J Exp Med (2015) 212:139–48. doi: 10.1084/jem.20140559 PMC4322048 25601652

[14] Finn RS Qin S Ikeda M Galle PR Ducreux M Kim TY. Atezolizumab Plus Bevacizumab in Unresectable Hepatocellular Carcinoma. N Engl J Med (2020) 382:1894–905. doi: 10.1056/NEJMoa1915745 32402160

[15] Finn RS Zhu AX. Targeting Angiogenesis in Hepatocellular Carcinoma: Focus on VEGF and Bevacizumab. Expert Rev Anticancer Ther (2009) 9:503–9. doi: 10.1586/era.09.6 19374603

[16] Caruso D Polici M Zerunian M Pucciarelli F Guido G Polidori T. Radiomics in Oncology, Part 1: Technical Principles and Gastrointestinal Application in CT and MRI. Cancers (Basel) (2021) 13:2522. doi: 10.3390/cancers13112522 34063937 PMC8196591

[17] Xu X Zhang HL Liu QP Sun SW Zhang J Zhu FP. Radiomic Analysis of Contrast-Enhanced CT Predicts Microvascular Invasion and Outcome in Hepatocellular Carcinoma. J Hepatol (2019) 70:1133–44. doi: 10.1016/j.jhep.2019.02.023 30876945

[18] Masokano IB Liu W Xie S Marcellin DFH Pei Y Li W. The Application of Texture Quantification in Hepatocellular Carcinoma Using CT and MRI: A Review of Perspectives and Challenges. Cancer Imaging (2020) 20:67. doi: 10.1186/s40644-020-00341-y 32962762 PMC7510095

[19] Yang L Gu D Wei J Yang C Rao S Wang W. A Radiomics Nomogram for Preoperative Prediction of Microvascular Invasion in Hepatocellular Carcinoma. Liver Cancer (2019) 8:373–86. doi: 10.1159/000494099 PMC6873064 31768346

[20] Wang W Gu D Wei J Ding Y Yang L Zhu K. A Radiomics-Based Biomarker for Cytokeratin 19 Status of Hepatocellular Carcinoma With Gadoxetic Acid-Enhanced MRI. Eur Radiol (2020) 30:3004–14. doi: 10.1007/s00330-019-06585-y 32002645

[21] Sterzynska K Klejewski A Wojtowicz K Swierczewska M Andrzejewska M Rusek D. The Role of Matrix Gla Protein (MGP) Expression in Paclitaxel and Topotecan Resistant Ovarian Cancer Cell Lines. Int J Mol Sci (2018) 19:2901. doi: 10.3390/ijms19102901 PMC6213242 30257426

[22] Pilco-Janeta D de la Cruz Puebla M Soriano J Osorio M Caballero I Perez AC. Aberrant Expression of N-Glycolyl GM3 Ganglioside Is Associated With the Aggressive Biological Behavior of Human Sarcomas. BMC Cancer (2019) 19:556. doi: 10.1186/s12885-019-5743-9 31182063 PMC6558727

[23] Gan C Pierscianek D El Hindy N Ahmadipour Y Keyvani K Sure U. The Predominant Expression of Cancer Stem Cell Marker ALDH1A3 in Tumor Infiltrative Area Is Associated With Shorter Overall Survival of Human Glioblastoma. BMC Cancer (2020) 20:672. doi: 10.1186/s12885-020-07153-0 32680476 PMC7368792

[24] Sun Z Li Y Wang Y Fan X Xu K Wang K. Radiogenomic Analysis of Vascular Endothelial Growth Factor in Patients With Diffuse Gliomas. Cancer Imaging (2019) 19:68. doi: 10.1186/s40644-019-0256-y 31639060 PMC6805458

[25] Van Griethuysen JJM Fedorov A Parmar C Hosny A Aucoin N Narayan V. Computational Radiomics System to Decode the Radiographic Phenotype. Cancer Res (2017) 77:e104–7. doi: 10.1158/0008-5472.CAN-17-0339 PMC5672828 29092951

[26] Zhang G Xu L Zhao L Mao L Li X Jin Z. CT-Based Radiomics to Predict the Pathological Grade of Bladder Cancer. Eur Radiol (2020) 30:6749–56. doi: 10.1007/s00330-020-06893-8 32601949

[27] Fan Y Yu Y Wang X Hu M Hu C. Radiomic Analysis of Gd-EOB-DTPA-Enhanced MRI Predicts Ki-67 Expression in Hepatocellular Carcinoma. BMC Med Imaging (2021) 21:100. doi: 10.1186/s12880-021-00633-0 34130644 PMC8204550

[28] Luo D Li H Hu J Zhang M Zhang S Wu L. Development and Validation of Nomograms Based on Gamma-Glutamyl Transpeptidase to Platelet Ratio for Hepatocellular Carcinoma Patients Reveal Novel Prognostic Value and the Ratio Is Negatively Correlated With P38MAPK Expression. Front Oncol (2020) 10:548744. doi: 10.3389/fonc.2020.548744 33344225 PMC7744698

[29] Gu D Xie Y Wei J Li W Ye Z Zhu Z. MRI-Based Radiomics Signature: A Potential Biomarker for Identifying Glypican 3-Positive Hepatocellular Carcinoma. J Magn Reson Imaging (2020) 52:1679–87. doi: 10.1002/jmri.27199 32491239

[30] Zhang X Wu Z Peng Y Li D Jiang Y Pan F. Correlationship Between Ki67, VEGF, and P53 and Hepatocellular Carcinoma Recurrence in Liver Transplant Patients. BioMed Res Int (2021) 2021:6651397. doi: 10.1155/2021/6651397 33954191 PMC8064788

[31] Ng F Kozarski R Ganeshan B Goh V. Assessment of Tumor Heterogeneity by CT Texture Analysis: Can the Largest Cross-Sectional Area Be Used as an Alternative to Whole Tumor Analysis? Eur J Radiol (2013) 82:342–8. doi: 10.1016/j.ejrad.2012.10.023 23194641

[32] Gillies RJ Kinahan PE Hricak H. Radiomics: Images Are More Than Pictures, They Are Data. Radiology (2016) 278:563–77. doi: 10.1148/radiol.2015151169 PMC4734157 26579733

[33] Granito A Galassi M Piscaglia F Romanini L Lucidi V Renzulli M. Impact of Gadoxetic Acid (Gd-EOB-DTPA)-Enhanced Magnetic Resonance on the Non-Invasive Diagnosis of Small Hepatocellular Carcinoma: A Prospective Study. Aliment Pharmacol Ther (2013) 37:355–63. doi: 10.1111/apt.12166 23199022

[34] Schulze K Nault JC Villanueva A. Genetic Profiling of Hepatocellular Carcinoma Using Next-Generation Sequencing. J Hepatol (2016) 65:1031–42. doi: 10.1016/j.jhep.2016.05.035 27262756

[35] Li S Peck-Radosavljevic M Ubl P Wadsak W Mitterhauser M Rainer E. The Value of [(11)C]-Acetate PET and [(18)F]-FDG PET in Hepatocellular Carcinoma Before and After Treatment With Transarterial Chemoembolization and Bevacizumab. Eur J Nucl Med Mol Imaging (2017) 44:1732–41. doi: 10.1007/s00259-017-3724-2 PMC5537334 28555333

[36] Li Z Si G Jiao DC Han X Zhang W Li Y. Portal Vein Stenting Combined With (125)I Particle Chain Implantation Followed by As2O3 in the Treatment of Hepatocellular Carcinoma With Portal Vein Tumour Thrombus. BioMed Res Int (2020) 2020:4109216. doi: 10.1155/2020/4109216 32090088 PMC7013352

[37] Montal R Andreu-Oller C Bassaganyas L Esteban-Fabro R Moran S Montironi C. Molecular Portrait of High Alpha-Fetoprotein in Hepatocellular Carcinoma: Implications for Biomarker-Driven Clinical Trials. Br J Cancer (2019) 121:340–3. doi: 10.1038/s41416-019-0513-7 PMC6738090 31285588

[38] Zhang C Wang N Tan HY Guo W Chen F Zhong Z. Direct Inhibition of the TLR4/MyD88 Pathway by Geniposide Suppresses HIF-1alpha-Independent VEGF Expression and Angiogenesis in Hepatocellular Carcinoma. Br J Pharmacol (2020) 177:3240–57. doi: 10.1111/bph.15046 PMC7312435 32144747

[39] Zhang L Kuang S Chen J Zhang Y Zhao B Peng H. The Role of Preoperative Dynamic Contrast-Enhanced 3.0-T MR Imaging in Predicting Early Recurrence in Patients With Early-Stage Hepatocellular Carcinomas After Curative Resection. Front Oncol (2019) 9:1336. doi: 10.3389/fonc.2019.01336 31850221 PMC6892896

[40] Feng X Tustison NJ Patel SH Meyer CH. Brain Tumor Segmentation Using an Ensemble of 3D U-Nets and Overall Survival Prediction Using Radiomic Features. Front Comput Neurosci (2020) 14:25. doi: 10.3389/fncom.2020.00025 32322196 PMC7158872

[41] Sun XF Zhang H. Clinicopathological Significance of Stromal Variables: Angiogenesis, Lymphangiogenesis, Inflammatory Infiltration, MMP and PINCH in Colorectal Carcinomas. Mol Cancer (2006) 5:43. doi: 10.1186/1476-4598-5-43 17026740 PMC1618857

[42] Granito A Forgione A Marinelli S Renzulli M Ielasi L Sansone V. Experience With Regorafenib in the Treatment of Hepatocellular Carcinoma. Therap Adv Gastroenterol (2021) 14:17562848211016959. doi: 10.1177/17562848211016959 PMC8165525 34104211

