# Supplementary material for: A Potential Prognostic Marker for Recognizing VEGF-Positive Hepatocellular Carcinoma Based on Magnetic Resonance Radiomics Signature
Source: Front Oncol. 2022 Apr 4;12:857715. doi: 10.3389/fonc.2022.857715 (PMC9013965; doi:10.3389/fonc.2022.857715)
Supplement: Supplementary file 1 [file DataSheet_1.docx]

Supplementary Material

**Methods 1: MRI protocol.**

All of the participants were examined on a Philips 3.0-T scanner (Philips Medical Systems, Netherlands). The Gd-EOB-DTPA-enhanced MR images were obtained in a 3.0-T scanner (Philips Medical Systems, Netherlands). Baseline sequences included diffusion-weighted imaging (DWI), T2-weighted phase, pre-contrast T1-weighted phase, dynamic T1-weighted arterial phase, portal venous phase, balanced phase, delayed phase, and hepatobiliary phase (HBP). A prototype single-shot spin echo echo-planar free-breathing DWI sequence using repetition time (TR), 985 milliseconds (ms);echo time (TE), 53 ms; field of view (FOV), 450×367 mm; matrix size, 152×120; and slice thickness, 6 mm was acquired before the dynamic examination. A corresponding apparent diffusion coefficient (ADC) map was automatically calculated with two b values of 0 and 800 s/mm^2^. For dynamic T1-weighted examination, we used the parameters TR, 3.0 ms; TE, 1.42 ms; flip angle, 10°; FOV , 375×280mm; matrix size, 252 × 184; and slice thickness, 3 mm. After an intravenous injection of 0.025 mmol/kg of gadoxetic acid (Primovist, Bayer) at a speed of 2 mL/s, we obtained images of arterial phase, portal venous phase (PVP), balanced phase, delayed phase, and HBP in 20-30 s, 60-70 s, 120 s ,180 s, and 20 min, respectively. T2-weighted images (TR, 1250ms; TE, 70 ms; FOV, 375 ×297 mm; matrix size, 268×186; slice thickness, 6 mm; gap, 2mm; flip angle, 90°) were obtained during the interval between delayed phase and hepatobiliary phase.

**Methods 2: immunohistochemical protocol.**

Tumor samples were obtained from non-necrotic HCC central areas. The VEGF was assessed using paraffin-embedded tissue samples that were cut into 3-μm slices. VEGF staining was performed according to an immunohistochemical staining standard protocol. Briefly, slides were baked for 1 hour at 60℃ and deparaffinized in xylene and rehydrated through a graded series of ethanol solutions, and then washed three times in PBS for 5 min per wash. After dewaxed into water, the sections were placed in the citrate repair solution, repaired under high pressure for 3 min, and slowly cooled to room temperature. The sections were stained with rabbit anti-VEGF polyclonal antibody (Wuhan Sanying Biotechnology, Wuhan, China) at a diluted ratio of 1:300 in humidified chamber overnight at 4℃. Secondary goat anti-rabbit antibody (Beijing Zhongshan Golden Bridge Biotechnology Company, China) was incubated at room temperature for 30 min. VEGF expression was visualized using 3,3’-diaminobenzidine (DAB), followed by counter-staining with hematoxylin.

**Table S1** Interobserver agreement of morphologic MR features between the two observers.

| Morphologic MR features | Observer 1 | Observer 2 | Interobserver agreement (95%CI) |
| --- | --- | --- | --- |
| Irregular Margin on HBP |  |  | 0.807(0.732-0.891) |
| Absence | 85 | 84 |  |
| Presence | 117 | 118 |  |
| Enhancement pattern * |  |  | 0. 774(0.657-0.852) |
| Arterial enhancement with washout | 162 | 159 |  |
| No or minimal enhancement | 21 | 24 |  |
| Persistent enhancement | 13 | 14 |  |
| Progressive enhancement | 6 | 5 |  |
| Arterial rim enhancement |  |  | 0.797 (0.709-0.879) |
| Presence | 132 | 125 |  |
| Absence | 70 | 77 |  |

* Inter-observer agreement was calculated using kappa test.

**Table S2. Detailed information of the selected features in hepatobiliary phase**

| **Feature name** | **Formula** | **Content** |
| --- | --- | --- |
| lbp-2D_glcm_Imc2 | Informational Measure of Correlation (IMC) 2 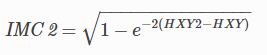 | IMC2 also assesses the correlation between the probability distributions of ii and jj (quantifying the complexity of the texture). Of interest is to note that HXY1=HXY2 and that HXY2−HXY≥0 represents the mutual information of the 2 distributions. Therefore, the range of IMC2 = [0, 1), with 0 representing the case of 2 independent distributions (no mutual information) and the maximum value representing the case of 2 fully dependent and uniform distributions (maximal mutual information, equal to log2(Ng)). In this latter case, the maximum value is 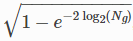 then equal to ,approaching 1. |
| original_shape_Elongation | Elongation 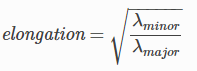 | Elongation shows the relationship between the two largest principal components in the ROI shape. For computational reasons, this feature is defined as the inverse of true elongation.  Here,λmajor and λminor are the lengths of the largest and second largest principal component axes. The values range between 1 (where the cross section through the first and second largest principal moments is circle-like (non-elongated)) and 0 (where the object is a maximally elongated: i.e. a 1 dimensional line). |
| square_glrlm_RunEntropy | Run Entropy (RE) 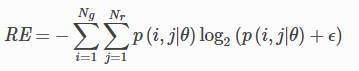 | Here, ϵ is an arbitrarily small positive number (≈2.2×10^−16^). RE measures the uncertainty/ randomness in the distribution of run lengths and gray levels. A higher value indicates more heterogeneity in the texture patterns. |
| wavelet-LHH_glcm_JointAverage | Joint Average 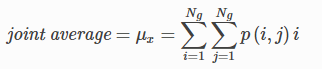 | Returns the mean gray level intensity of the ii distribution. |
| wavelet-HHL_glcm_ClusterShade | Cluster Shade 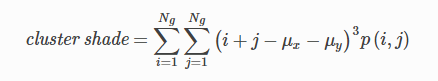 | Cluster Shade is a measure of the skewness and uniformity of the GLCM. A higher cluster shade implies greater asymmetry about the mean. |

**Table S3. Detailed information of the selected features in portal venous phase**

| **Feature name** | **Formula** | **Content** |
| --- | --- | --- |
| gradient_gldm_SmallDependenceLowGrayLevelEmphasis | SmallDependenceLowGrayLevelEmphasis**(SDLGLE)** | Measures the joint distribution of small dependence with lower gray-level values. |
| original_firstorder_Skewness | Skewness 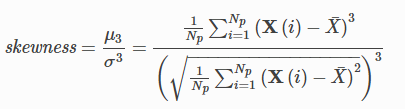 | Where μ_3_ is the 3^rd^ central moment. Skewness measures the asymmetry of the distribution of values about the Mean value. Depending on where the tail is elongated and the mass of the distribution is concentrated, this value can be positive or negative. |
| wavelet-LLH_firstorder_Range | Range 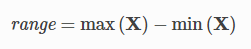 | The range of gray values in the ROI. |
| wavelet-LHL_firstorder_Skewness | Skewness 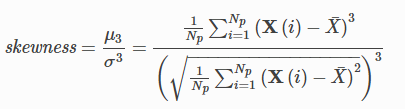 | Where μ_3_ is the 3^rd^ central moment. Skewness measures the asymmetry of the distribution of values about the Mean value. Depending on where the tail is elongated and the mass of the distribution is concentrated, this value can be positive or negative. |
| wavelet-HLH_glcm_Correlation | Correlation | Correlation is a value between 0 (uncorrelated) and 1 (perfectly correlated) showing the linear dependency of gray level values to their respective voxels in the GLCM |
| log-sigma-4-0-mm-3D_firstorder_Mean | Mean 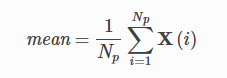 | The average gray level intensity within the ROI. |
| original_gldm_LargeDependenceLowGrayLevelEmphasis | Large Dependence Low Gray Level Emphasis (LDLGLE) 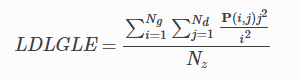 | Measures the joint distribution of large dependence with lower gray-level values. |
| wavelet-HLH_glszm_SmallAreaLowGrayLevelEmphasis | Small Area Low Gray Level Emphasis (SALGLE) 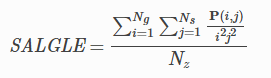 | SALGLE measures the proportion in the image of the joint distribution of smaller size zones with lower gray-level values. |

**Table S4**. Comparisons among AUCs between every two model.

| Delong test  (*P* value) | Combined model vs. Clinical model | Combined model vs. Fusion radiomics model | Fusion radiomics model vs. Clinical model | Combined model vs.Hepatobiliary phase | Combined model vs.Venous phase |
| --- | --- | --- | --- | --- | --- |
| Training dataset | <0.0001 | 0.0018 | 0.0003 | <0.0001 | <0.0001 |
| Test dataset | 0.0932 | 0.2133 | 0.3759 | 0.0752 | 0.1090 |

**Table S5.** Stratification analysis of the combined model for identifying VEGF-positive.

| Group | Training Dataset | | | | Test Dataset | | | |
| --- | --- | --- | --- | --- | --- | --- | --- | --- |
|  | AUC(95%CI) | ACC | SENS | SPEC | AUC(95%CI) | ACC | SENS | SPEC |
| Age (years) | | | | | | | | |
| ≤56  (N=110) | 0.965  (0.929-1.000) | 0.905 | 0.865 | 0.946 | 0.877  (0.751-1.000) | 0.806 | 0.722 | 0.889 |
| > 56  (N=92) | 0.884  (0.800-0.968) | 0.868 | 0.862 | 0.872 | 0.757  (0.522-0.993) | 0.583 | 0.700 | 0.500 |
| Gender | | | | | | | | |
| Male  (N=169) | 0.929  (0.884-0.975) | 0.861 | 0.855 | 0.868 | 0.813  (0.695-0.931) | 0.685 | 0.571 | 0.808 |
| Female  (N=33) | 0.923  (0.825-1.000) | 0.852 | 0.786 | 0.923 | 1.000  (1.000-1.000) | 0.833 | 0.750 | 1.000 |

**Note:** AUC area under the curve; CI confidence interval; ACC accuracy;SENS sensitivity;

SPEC specificity.

**Table S6.** The coefficients and relative weights of 13 selected radiomics features of logistic regression in combined model.

| **Feature** | **coefficient** | **relative_weight** |
| --- | --- | --- |
| original_shape_Elongation_HBP | 0.7352 | 0.714 |
| gradient_gldm_SmallDependenceLowGrayLevelEmphasis_P | 0.7188 | 0.6981 |
| wavelet-HLH_glszm_SmallAreaLowGrayLevelEmphasis_P | 0.7029 | 0.6826 |
| lbp-2D_glcm_Imc2_HBP | 0.4435 | 0.4307 |
| wavelet-LHL_firstorder_Skewness_P | 0.3592 | 0.3489 |
| wavelet-LHH_glcm_JointAverage_HBP | -0.2049 | -0.199 |
| square_glrlm_RunEntropy_HBP | -0.3277 | -0.3183 |
| log-sigma-4-0-mm-3D_firstorder_Mean_P | -0.5871 | -0.5702 |
| original_firstorder_Skewness_P | -0.6731 | -0.6537 |
| wavelet-HLH_glcm_Correlation_P | -0.7971 | -0.7742 |
| wavelet-LLH_firstorder_Range_P | -0.836 | -0.8119 |
| wavelet-HHL_glcm_ClusterShade_HBP | -0.9568 | -0.9292 |
| original_gldm_LargeDependenceLowGrayLevelEmphasis_P | -1.0297 | -1 |

Note: Feature values are denominated in four lays: The first-lay names indicate whether or not the filter is employed, original names indicate that the filter is not utilized; log: Laplacian of Gaussian filter; **lbp-2D**: calculates and returns a local binary pattern applied in 2D; **gradient**: returns the gradient magnitude; wavelet represents the wavelet transform; **square**: takes the square of the image intensities and linearly scales them back to the original range. The second layer is divided into three categories: shape, first-order, texture (glszm, glcm, gldm, glrlm). The third layer is the name of the specific features. The fourth layer is the name of the specific phase of the MRI, P stands for portal venous phase and HBP stands for hepatobiliary phase.

**Figure S1. Radiomics features stability.**


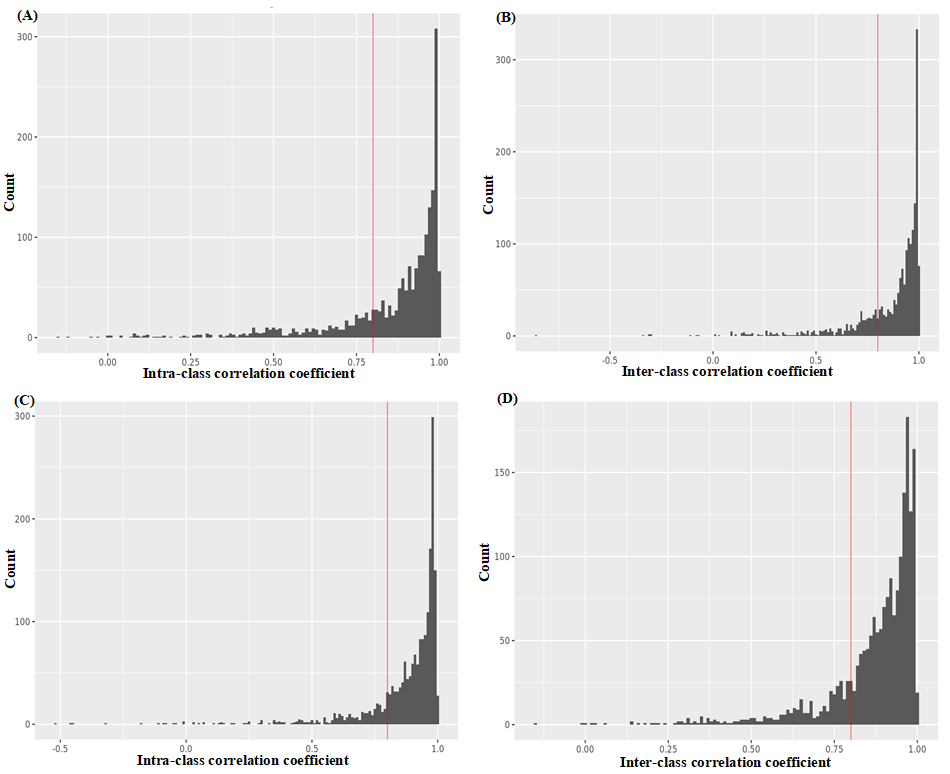


**Figure S1**. Histogram of the Intra- and Inter-class correlation coefficient (ICCs). The intra and inter-class correlation coefficient (ICCs) was used to determine the stability of the features. The features with the ICCs greater than 0.8 were included for the further analysis. After reproducibility test, (A) 1472 of the initial 1906 MR image features in hepatobiliary phase were considered robust in Intra-class correlation analysis; (B) 1475 of the initial 1906 MR image features in hepatobiliary phase were considered robust in Inter-class correlation; (C) 1575 of the initial 1906 MR image features in portal venous phase were considered robust in Intra-class correlation analysis. (D) 1549 of the initial 1906 MR image features in portal venous phase were considered robust in Inter-class correlation analysis.

**Figure S2. Correlation heat map of the selected 13 features in the combined model.**


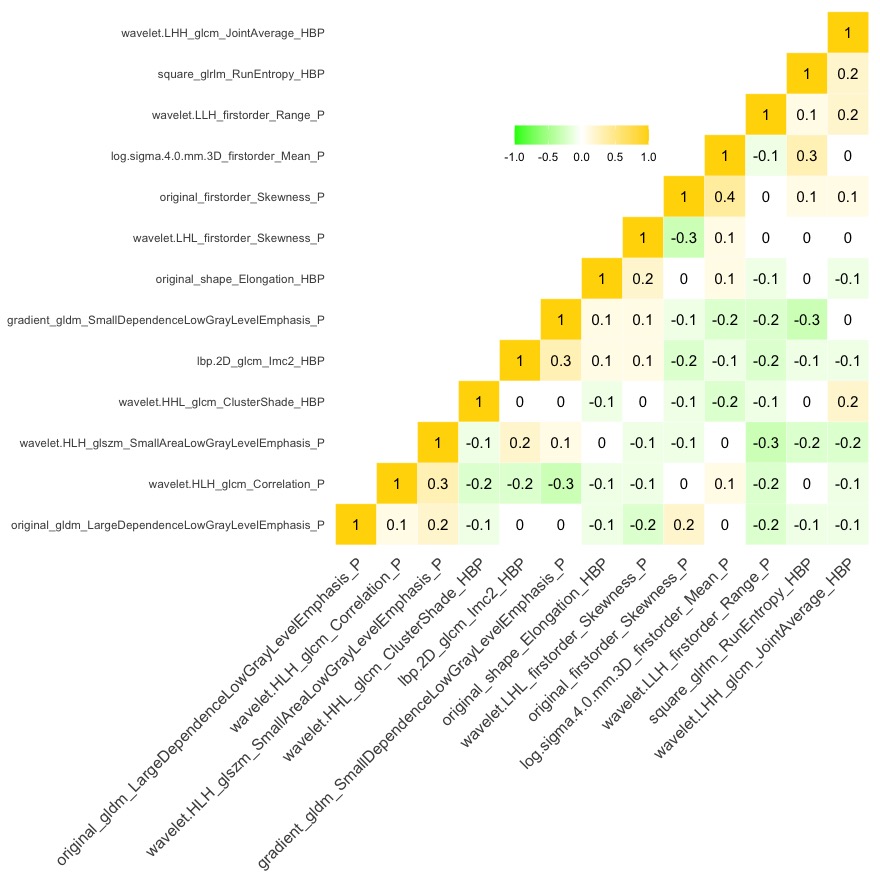


**Figure S2**. **Correlation heat map of the selected 13 features** **in the combined model. All the features’ correlation coefficients are less than 0.5.**
